# Stabilization and improved functionality of three-dimensional perfusable microvascular networks in microfluidic devices under macromolecular crowding

**DOI:** 10.1186/s40824-023-00375-w

**Published:** 2023-04-19

**Authors:** Ho-Ying Wan, Jack Chun Hin Chen, Qinru Xiao, Christy Wingtung Wong, Boguang Yang, Benjamin Cao, Rocky S. Tuan, Susan K. Nilsson, Yi-Ping Ho, Michael Raghunath, Roger D. Kamm, Anna Blocki

**Affiliations:** 1 Institute for Tissue Engineering and Regenerative Medicine, The Chinese University of Hong Kong, Hong Kong SAR, China; 2 School of Biomedical Sciences, Faculty of Medicine, The Chinese University of Hong Kong, Hong Kong SAR, China; 3 Department of Biomedical Engineering, Faculty of Engineering, The Chinese University of Hong Kong, Hong Kong SAR, China; 4 Department of Orthopaedics & Traumatology, Faculty of Medicine, The Chinese University of Hong Kong, Hong Kong SAR, China; 5 Biomedical Manufacturing Commonwealth Scientific and Industrial Research Organisation (CSIRO), Melbourne, Australia; 6 Australian Regenerative Medicine Institute, Monash University, Melbourne, Australia; 7 Institute for Chemistry and Biotechnology, Zurich University of Applied Sciences, Wädenswil, Switzerland; 8 Department of Biology and Mechanical Engineering, Massachusetts Institute of Technology, Cambridge, MA USA; 9 Center for Neuromusculoskeletal Restorative Medicine (CNRM), Hong Kong Science Park, Shatin, New Territories, Hong Kong SAR China

**Keywords:** Microvascular networks, Microfluidic device, Macromolecular crowding, Vessel retraction, Basement membrane, Vascular barrier function

## Abstract

**Background:**

There is great interest to engineer *in vitro* models that allow the study of complex biological processes of the microvasculature with high spatiotemporal resolution. Microfluidic systems are currently used to engineer microvasculature *in vitro*, which consists of perfusable microvascular networks (MVNs). These are formed through spontaneous vasculogenesis and exhibit the closest resemblance to physiological microvasculature. Unfortunately, under standard culture conditions and in the absence of co-culture with auxiliary cells as well as protease inhibitors, pure MVNs suffer from a short-lived stability.

**Methods:**

Herein, we introduce a strategy for stabilization of MVNs through macromolecular crowding (MMC) based on a previously established mixture of Ficoll macromolecules. The biophysical principle of MMC is based on macromolecules occupying space, thus increasing the effective concentration of other components and thereby accelerating various biological processes, such as extracellular matrix deposition. We thus hypothesized that MMC will promote the accumulation of vascular ECM (basement membrane) components and lead to a stabilization of MVN with improved functionality.

**Results:**

MMC promoted the enrichment of cellular junctions and basement membrane components, while reducing cellular contractility. The resulting advantageous balance of adhesive forces over cellular tension resulted in a significant stabilization of MVNs over time, as well as improved vascular barrier function, closely resembling that of *in vivo* microvasculature.

**Conclusion:**

Application of MMC to MVNs in microfluidic devices provides a reliable, flexible and versatile approach to stabilize engineered microvessels under simulated physiological conditions.

**Supplementary Information:**

The online version contains supplementary material available at 10.1186/s40824-023-00375-w.

## Background

Microvessels act as the ultimate barrier between tissue and blood and regulate the exchange of molecules and cells [1]. Endothelial cells, which form the inner lining of microvessels, create this semipermeable barrier via a combination of cellular junctions, their glycocalyx, and the basement membrane that consists of vessel-specific extracellular matrix (ECM) [2]. Embedded within the basement membrane and in direct contact with endothelial cells are pericytes, which are crucial for microvessel maturation, maintenance, as well as proper function. Pericytes have also been reported to be in essence mesenchymal stem cells (MSCs) [1, 3].

The microvascular system and especially endothelial cells play a major role in inflammation, act as an endocrine organ and tightly regulate blood coagulation [1]. Hence, the microvasculature plays a pivotal role in many physiological and pathophysiological processes including cancer and cardiovascular diseases [1, 4] and is an active area of biomedical research. Traditionally, processes involving microvasculature have been investigated either *in vivo* or in 2-dimensional (2D) *in vitro* models. Unfortunately, animal models are often limited by low resolution during real-time monitoring and interspecies differences, which can hamper clinical translation [5]. On the other hand, 2D monolayer systems lack the 3D context relevant for proper function of blood vessels [6].

There is thus a recognized need to engineer functional microvascular networks (MVNs) *in vitro* that are able to recapitulate physiological properties as closely as possible [7]. Previous MVN microfabrication approaches include 3D printing of precisely organized channel structures [8], channel-molded hydrogels [9] and microfluidic channels that could be seeded with an endothelial monolayer [10]. In addition, approaches to directly “seed” microvasculature by cell sheet stacking or 3D bio-printing of cells were also employed [11]. These approaches have enabled the study of microvessel-like structures and their properties *in vitro*, such as vascular permeability [12] and response to shear stress/fluid flow [13, 14], VEGF [15], cyclic AMP [16] and mechanical stimulation [17]. However, these microvessel-like structures possess a limited ability to reconstitute the characteristic features of the *in vivo* endothelium, such as physiologically representative vascular barrier functions [18, 19].

**Fig. 1:**
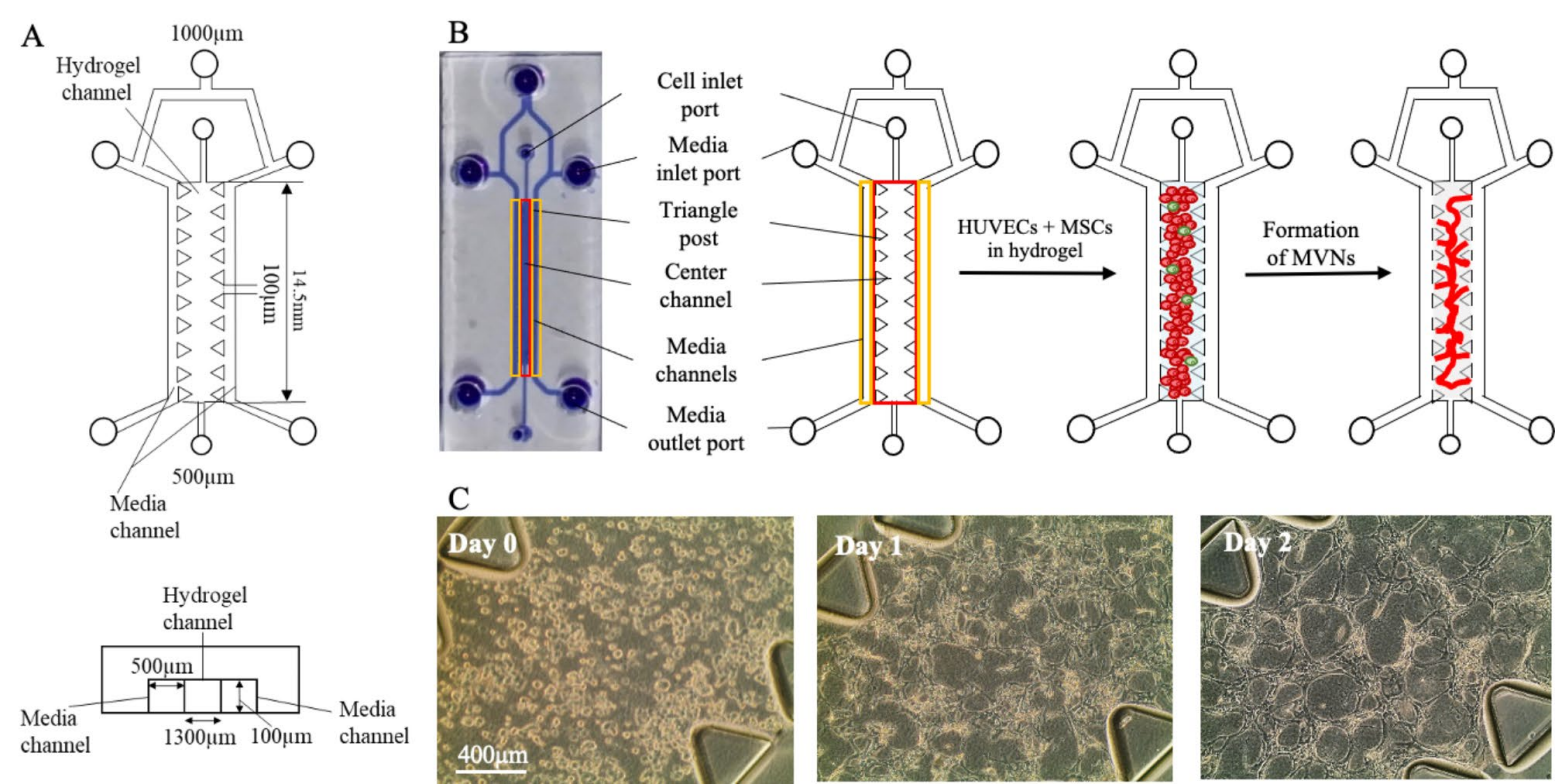
HUVECs and MSCs form interconnected MVNs within the microfluidic device. (A) Device dimensions of the three-channel microfluidic device. (B) Photograph and schematic of cell seeding into the three-channel microfluidic device. HUVECs and MSCs are seeded in a fibrinogen solution into the center channel via the cell inlet port. The co-supplemented thrombin induces fibrin polymerization within 20 min. Over the next two days, seeded cells re-arrange into interconnected MVNs within the fibrin hydrogel. (C) Representative phase contrast micrographs of seeded HUVECs and MSCs forming MVNs in microfluidic devices within 2 days. Scale bar = 400 μm

Hence, recent efforts have focused on engineering perfusable MVNs with *in vivo*-like cell morphology and physiologically representative functions [19–25]. Microfluidic-based approaches present great promise for this purpose, as they are easy to fabricate, and their design can be adjusted specifically for a desired application and to study the process of interest in a controlled environment in detail and over time [19, 21–24]. In particular, by using a parallel multichannel set-up within microfluidic devices, endothelial cells can be seeded with various other cell types in biologically derived hydrogels (e.g. fibrin) into the center channel [22]. The hydrogel region is flanked by channels of culture medium, separated from the hydrogel channels by trapezoidal post arrays, which provide enough surface tension to fill the inner chamber with the hydrogel [19]. The spaces between the trapezoidal posts allow for a direct hydrogel-medium interface, critical for gas exchange and delivery of nutrients during the culture period. The endothelial cells within the hydrogel then spontaneously undergo physiological morphogenesis (vasculogenesis), resulting in the *de novo* formation of interconnected MVNs with patent lumina and openings towards the media channels (Fig. 1A) [23]. Importantly, formation of MVNs via vasculogenesis appears to be a key requirement, as endothelial cells, after undergoing this process, produce MVNs with improved barrier-properties, interstitial flow regulation, *in vivo*-like cell morphology and thus more physiologically representative functions [20, 26, 27]. Vessel parameters such as diameter, branching, as well as stability (to a certain degree) can be regulated by hydrogel concentration, endothelial cell seeding density, co-seeding with perivascular cells (e.g. pericytes/MSCs), interstitial flow as well as signaling factors and importantly protease inhibitors [22, 27–29]. However, MVNs cultured under standard culture conditions in microfluidic devices exhibit a very limited stability. As a result, the utilization of these MVNs for long-term biological investigations is greatly restricted. As shown recently by some of us, the stability of MVN is particularly dependent on the batch of human umbilical cord endothelial cells (HUVECs) acquired [30]. This can be further improved when these cells are immortalized [30]. However, since the majority of HUVECs form MVNs with a limited stability, this remains a major hurdle for most investigations.

Longer lasting stabilization of perfusable MVNs could be achieved by direct [31] or indirect co-culture of MVNs with stromal cells such as fibroblasts, where MVN stabilization occurs through fibroblast-derived paracrine and juxtacrine factors [20, 22, 32]. Indeed, studies involving MVNs stabilized by fibroblast paracrine factors were reported to allow for continuous experiments for 1 [20, 22, 24, 26] to 2 weeks [32]. However, this approach leads to a higher complexity of the system, thus confounding data interpretation, and resulting in a higher variability due to batch-to-batch differences of the cells utilized. Furthermore, the overproportioned presence of fibroblasts [33] can unbalance more delicate biological systems, such as engineered stem cell niches, or lead to non-physiologically representative responses. It is also noteworthy that all of the above approaches still require the utilization of protease inhibitors, to minimize premature destabilization of MVNs [22]. These agents can interfere with cellular processes, contributing to non-physiological responses [34, 35].

In view of these limitations, we proposed to introduce macromolecular crowding (MMC) to human MVNs in microfluidic devices in order to stabilize MVNs and improve their functionality without the requirement for the over-proportional presence of auxiliary cells (fibroblasts) and protease inhibitors. MMC occurs naturally in the physiologically “crowded” *in vivo* environment of tissues. The biophysical principle of MMC is based on macromolecules occupying space, thereby increasing the effective concentration of other components, as well as the thermodynamic activity of the system [36]. As a result, protein folding, intermolecular interactions, as well as enzyme and reaction kinetics are enhanced. MMC has been applied to cell culture by introducing carbohydrate-based macromolecules at a physiologically relevant fractional volume occupancy (FVO) into culture medium [3, 37–44]. Applied MMC accelerated various biological processes including ECM assembly *in vitro*. While these studies have been reported in 2-dimensional (2D) cultures [40, 45–48], a recent study on crowding of spheroid cultures has shown that MMC also enhanced ECM deposition in 3-dimensional (3D) cultures [41, 49]. We thus hypothesized that MMC will facilitate the formation of a robust vascular basement membrane, thereby stabilizing human MVNs and improving their functionality *in vitro*.

## Materials and methods

### Device fabrication

Microfluidic devices were fabricated by replica molding on a silicon wafer and soft lithography using PDMS (polydimethylsiloxane), as described previously [22]. Briefly, a 100 μm layer of SU-8 3050 negative photoresist (Kayaku Advanced Materials, Massachusetts, USA) primer was spin-coated on a silicon wafer before exposed to a photomask exhibiting the negative pattern of the channel structures designed by computer aided designs (CAD) for photolithography. The SU8 was then exposed to UV light (set as 20 mW/cm^2^ at 365 nm) for 45 s, followed by the pattern developing. PDMS and curing agent (Sylgard 184, Dow Corning, Michigan, USA) were mixed at 10:1 (W/W) ratio and cast onto the SU8 master. After thermal curation at 60˚C for two hours, a positive replica-molded pattern on PDMS was separated from the wafer. Patterned PDMS was cut into individual devices and inlet and outlet ports were punched using 1 and 3 mm biopsy punchers. Next, the devices and glass slides were cleaned with 100% ethanol, water, and dried with a nitrogen gas air gun before being treated with oxygen plasma (Harrick Plasma, New York, USA) for 45 s to create covalent bonding between the glass slide and the PDMS device. Right after the PDMS pieces and glass slides were assembled, the channels were coated with 1 mg/ml of poly-L-lysine (PLL) (Molecular weight: 30,000–70,000) (Meryer, Shanghai, China, Cat#. 25988-63-0) dissolved in water for at least 20 min before autoclaving. PLL coating increased the hydrophilicity of the device enabling easy loading of the hydrogel.

### Device design

A previously established microfluidic design with a 3 channels system as displayed in Fig. 1A was utilized [22]. The channels have a height of 100 μm and length of 14.5 mm with triangular posts separating the center hydrogel channel from the adjacent media channels. The posts are 100 μm apart. The width of the hydrogel channel and media channels is 1300 μm and 500 μm, respectively. Media inlet ports and cell inlet ports have a diameter of 1000 μm and 500 μm, respectively.

### Cell culture

Primary HUVECs (pooled) (ATCC, Cat#. PCS-100-013) and GFP-expressing HUVECs (TTFLUOR HUVECs) (Innport, Primera Planta, Spain Cat#. P20201) were cultured in Endothelial Growth Medium (EGM-2) (Lonza, Walkersville, MD, USA, Cat#. CC3162). Human bone marrow derived MSCs (Milipore, Temecula, CA, USA, Cat#. SCC034) were cultured in Dulbecco’s Modified Eagle Medium (DMEM) (Gibco, Life Technologies, Grand Island, NY, USA, Cat#. 10567-014) supplemented with 10% fetal bovine serum (FBS) (Gibco, Life Technologies, Cat#. 16,000,044), as well as 1% of 100 U/mL penicillin and 100 µg/mL streptomycin (P/S) (Gibco, Life Technologies, Cat#. 15140-122). Immortalized Human Bone Marrow Mesenchymal Cells – hTERT (hTERT-MSCs) (Applied Biological Materials (abm) Inc., Richmond, BC, Canada, Cat#. T0523) were cultured in Roswell Park Memorial Institute (RPMI) 1640 Medium (Gibco, Life technologies, Cat#. 11,875,093) with 10% of fetal bovine serum (FBS) (Hyclone, Cytiva, Marlborough, MA, USA, Cat#. SH30084.03), 1% of 100 U/mL penicillin and 100 µg/mL streptomycin (P/S) (Gibco, Life Technologies, Cat#. 15140-122) and GlutaMAX™ Supplement (Gibco, Life technologies, Cat#. 35,050,061). hTERT-MSCs were cultured with HUVECs for permeability assay using Texas Red™ BSA. HUVECs and MSCs were cultured in tissue culture polystyrene flasks coated with 0.1% gelatin (Sigma-Aldrich, Saint Louis, MO, USA, Cat#. G1890) at 37 °C under 5% CO_2_. At ~ 80% confluency, all cells were trypsinized using TrypLE™ Express (Gibco, Life Technologies, Cat#. 12605-010) for 3 min at 37 °C and resuspended in EGM-2, DMEM with 10% FBS and 1% P/S and RPMI with 10% FBS and 1% P/S, respectively.

### PDMS device seeding

Fibrinogen powder (Sigma-Aldrich, Saint Louis, USA, Cat#. F8630-5G) was dissolved in phosphate buffered saline (PBS) at 15 mg/ml freshly for each experiment. Thrombin solution (Sigma-Aldrich, Saint Louis, USA, Cat#. T4648) was prepared in 1% (w/v) bovine serum albumin (BSA, Sigma-Aldrich, Cat#. A7906) in PBS solution at 100 U/ml, and stored in aliquots at -20 °C. HUVECs and MSCs (40:1) were resuspended in EGM-2 containing 6 U/ml of thrombin, the cell solution was mixed with fibrinogen solution at 1:1 ratio to have final concentrations at 7.5 mg/ml for fibrinogen, 3 U/ml for thrombin, 6 × 10^6^ cells/ml HUVECs and 1.5 × 10^5^ cells/ml for MSCs or hTERT MSCs, respectively. The mixture was then quickly introduced into the center channel and the device was placed at 37 °C in a humidified incubator for 15 min to allow the fibrinogen to be polymerized into a fibrin hydrogel by thrombin. Next, EGM-2 was added into the medium channels. Medium was changed on a daily basis and MMC (25 mg/ml Ficoll 400 (Cytiva, Marlborough, MA, USA, Cat#. 17-0300-50) and 37.5 mg/ml of Ficoll 70 (Cytiva, Marlborough, MA, USA, Cat#. 17-0210-10)) containing medium was optionally introduced from day 1 onwards. The optimal concentration of the Ficoll mixture was optimized previously, exhibits a calculated fractional volume occupancy (FVO) of 17% and was demonstrated to enhance ECM assembly and deposition [39].

For permeability test, one of the medium channels was seeded with monolayer of endothelial cells on day 2 of culture. In brief, after gel formation on day 0 of culture, both medium channels were coated with 50 µg/ml of fibronectin solution (from bovine plasma) (Sigma Aldrich, Saint Louis, USA, Cat#. F1141-5MG) in PBS for 1 h at 37 °C before washing with EGM-2. On day 2 of culture, all media were removed from ports and 50 µl of the respective medium (EGM-2 or EGM-2 supplemented with MMC) was introduced to inlet ports for both channels until inlet and outlet ports reached equilibrium. Additional 20 µl of the respective medium was added into the inlet port at the medium channel to be seeded with endothelial cells before 10 µl of endothelial cells (10 × 10^6^ cells/ml) resuspended in their respective medium were injected into that medium channel through its inlet port. After 2 min, the same procedure was repeated at the outlet port. The additional medium created hydrostatic pressure to facilitate the attachment of endothelial cells to the gel interface. The cells were allowed to attach for 3 h before washing with their respective medium to remove non-attached cells.

For 2D cell layer cultures, HUVECs and MSCs were seeded at 7 × 10^3^ cells/ml per well into 48-well plates in their respective medium. Medium optionally supplemented with MMC was added on the next day.

### Immunostaining

After 4 days of culture, the devices were washed with PBS and fixed with 4% paraformaldehyde (PFA) (Thermo Scientific, Cat#. 5735) in PBS through the medium channels for 15 min, then permeabilized with 0.25% Triton X-100 in PBS for 10 min. After blocking with 5% BSA for 1 h, samples were incubated with primary antibodies (see Table 1) in PBS containing 0.5% BSA for 16 h at 4 °C. Samples were then washed three times with PBS for 5 min each, before being incubated with secondary antibodies (see Table 1) for at least 3 h at room temperature. For staining of 2D cell layers, cells were seeded on day 0 and changed into control or crowded medium on day 1. On day 2, 4% of PFA was used for fixation before phalloidin and DAPI (see Table 1) was added followed by a 1 h incubation at room temperature. The samples were washed and maintained in PBS.

**Table 1:** List of antibodies and other staining reagents

Reagents	Host	Dilution	Supplier	Cat#.
Primary antibodies				
Anti-CD31	Mouse	1:200 (ICC)	Abcam	ab9498
Anti-collagen IV	Rabbit	1:100 (ICC)	Abcam	ab6586
		1:500 (WB)		
Anti-Laminin	Mouse	1:100 (ICC)	Abcam	ab77175
		1:500 (WB)		
Anti-VE-cadherin	Mouse	1:200 (ICC)	Santa Cruz	sc-9989
		1:500 (WB)		
Anti-β-catenin	Mouse	1:200 (ICC)	Santa Cruz	sc-7963
		1:500 (WB)		
Anti-Vinculin	Rabbit	1:200 (ICC)	Abcam	ab155120
Anti-GAPDH	Rabbit	1:500 (WB)	Abcam	ab181602
Secondary antibodies				
Anti-mouse-AF-555	Goat	1:200	Abcam	ab150118
Anti-rabbit-AF-488	Goat	1:200	Abcam	ab150077
Anti-mouse-AF-488	Goat	1:200	Abcam	ab150113
Anti-rabbit-HRP	Goat	1:6000	Abcam	ab6721
Anti-mouse-HRP	Goat	1:5000	Abcam	ab205719
Others				
Phalloidin-AF 555		1:500	Abcam	ab176756
DAPI*		1:500	Thermo fisher	62,247

*DAPI, 4′,6-diamidino-2-phenylindole; ICC, Immunocytochemistry; WB, Western blotting

### Microcopy

For confocal imaging, images were acquired with a Nikon C2 + point scanning confocal microscope (Nikon, Tokyo, Japan) at 10X, 20X and 60X magnification. Images of collagen IV, laminin, VE-cadherin and β-catenin are for qualitative analysis only, as exposure time was adjusted for each image to ensure optimal visibility. MVNs formed by GFP-HUVECs were monitored for 10 days using an Olympus IX70 inverted fluorescence microscope (Olympus, Tokyo, Japan) equipped with LED illuminator (pE-300^white^, CoolLED) and SPOT 5.4 BASIC Software (SPOT Imaging). Collagen gels and 2D cell layers were imaged using an Olympus IX83 inverted fluorescence microscope (Olympus, Tokyo, Japan) equipped with CellSense Dimension image acquisition software (Olympus, Tokyo, Japan). All images were analyzed with ImageJ software (https://imagej.nih.gov/ij/).

### Quantification of MVN vascular junction and tubule length per field of view (FOV)

MVNs were analyzed using ImageJ software (https://imagej.nih.gov/ij/) and Angiogenesis Analyzer plugin (https://imagej.nih.gov/ij/macros/toolsets/Angiogenesis%20Analyzer.txt). Briefly, raw images were converted into binary images using automated thresholds for binary tree analysis in the Angiogenesis Analyzer plugin. Number of junctions and total branching lengths were measured and presented as number of vascular junctions and tubule length per FOV, respectively. Junctions were denoted by points that had at least 3 neighbors, and tubule length referred to length of elements bound by two junctions or between one junction and one end point.

### Conjugation of FITC-PVP

Polyvinylpyrrolidone (PVP, 10 mg/ml; Sigma-Aldrich, Saint Louis, MO, USA, Cat#. PVP40) was added to 5-azido-2-nitrobenzoic acid N-hydroxysuccinimide ester (5-NABSIE, 2 mg/ml; Sigma-Aldrich, Saint Louis, MO, USA, Cat#. A3282) in DMSO yielding the equivalent of 1/75 of monomer units in the reaction batch. The resulting mixture was incubated under UV irradiation for 20 min, leading to 5-NABSIE activation, nitrene radical formation and C-H insertion into the PVP polymer strand, rendering the polymer amino reactive. In parallel, 10 mg/ml of fluorescein (FL) (Sigma-Aldrich) in 50% DMSO in water, equivalent to 1/7.5 monomer units related to PVP was mixed with equimolar amounts of carbonyldiimidazole (CDI) (Sigma-Aldrich, Saint Louis, MO, USA, Cat#. 115,533) (10 mg/ml in DMSO) and allowed to react for 10 min. The CDI activated FL was mixed at equimolar ratio with propylenediamine (PDA) (Sigma-Aldrich) solution (10 mg/ml in DMSO) under vigorous mixing and was allowed to react for 20 min. The amino reactive PVP solution was then mixed with the amino functional FL solution in a molar ratio of 1:10, having 10 times excess of amino functional FL compared to reactive groups on the PVP backbone. This solution was allowed to react for 3 h at room temperature followed by 1:3 dilution with water and dialysis against water for 3 days with dialysis medium change every 24 h. The resulting polymer solution was freeze-dried and kept as powder for specific use.

### Microvascular network permeability assay and quantification of permeability coefficient

MVN permeability was quantified by introducing 250 µg/ml of FITC-PVP or Texas Red™ Albumin from Bovine Serum (BSA) (Invitrogen, Cat#. A23017) in EGM-2 solution into one of the medium channels. For this, one medium channel was seeded with an endothelial monolayer on day 2. On day 4 of culture, medium was removed from each port and 15 µl of FITC-PVP solution was added into the outlet port of the channel seeded with endothelial cells. MVNs were then perfused by hydrostatic pressure resulting from the different levels of medium in the two medium channels until an equilibrium was reached. Time lapse images were taken every 15 s for 15 min using a Nikon Ti-E inverted fluorescent microscope (Nikon, Tokyo, Japan). Images were used to compute the permeability coefficient as described previously [12]. Briefly, the efflux rate of the fluorophores equals to the rate it accumulates in the hydrogel assuming the fluorescence intensity in the vessel is constant and the vessels are cylindrical. To calculate the permeability coefficient, measuring windows were drawn that included the vessel and the hydrogel embedded within. The windows were placed at vessels with a diameter smaller than 50 μm to ensure their circularity. This is of high importance as the formula for calculating the permeability coefficient is only valid with circular lumens. Furthermore, windows were placed at locations that did not exhibit any contamination by dye diffusing directly into the hydrogel from the side channel. The average fluorescence intensity at initial and final time points was obtained and the permeability was calculated according to the equation,

$$P \left(\frac{cm}{s}\right)=\frac{1}{{I}_{i}-{I}_{b}}\left(\frac{{I}_{f}-{I}_{i}}{{\Delta }t}\right)\times \frac{d}{4}$$

$\text{w}\text{h}\text{e}\text{r}\text{e} {I}_{i}$, ${I}_{b}$ and ${I}_{f}$ represent the average intensity in the measuring window at the initial time point, background, and final time point; $\varDelta t$ refers to the duration between initial and final time point; and $d$ is the average diameter of the vessel in the measuring window. n = 15 were obtained.

### Western blotting

PDMS devices were peeled off from the glass slide using a cutter and cells within the center channel were lysed using a 1:1 mixture of 2x Laemmli buffer and 2x protease inhibitor cocktail (Sigma-Aldrich, Cat#. P8340). Protein concentrations of collected samples were measured using a Bicinchoninic acid (BCA) Protein Assay Kit (Thermo Fisher, Cat#. A53226). Samples were denatured at 95 °C for 5 min and loaded at equal protein amounts into 8% SDS-polyacrylamide gels (Life Technologies, Cat#. HC2040) and subjected to electrophoresis at 120 V. After protein separation, samples were electrotransferred to a polyvinylidene difluoride membrane (Thermo Scientific, Cat#. 88,518) using a Power Blotter XL SYS (Life Technologies, Rockford, IL, USA, cat#0.34580). For membrane staining, membranes were incubated with 5% skimmed milk (Phygene Biotechnology Co Ltd, FuZhou, China, Cat#. PH1519) in TBS-Tween 20 (TBST), containing 50 mM Tris, 150 mM NaCl and 0.5% Tween 20 (Sigma-Aldrich, Cat#. P2287) to block non-specific antibody binding before incubation with primary antibodies (see Table 1) in TBST containing 1% skimmed milk at 4 °C overnight. After washing three times with TBST, secondary antibodies (see Table 1) resuspended in TBST containing 1% skimmed milk were added to the blots for 1 h at room temperature. Proteins bands were then detected with ECL Super Signal West Pico Plus (Life Technologies, Cat#34,580) using ChemiDocTM MP Imaging System (Bio-Rad Laboratories) and quantified by Image Lab 6.1 software (Bio-Rad, https://www.bio-rad.com/en-hk/product/image-lab-software?ID=KRE6P5E8Z).

### Cellular contractility assay

HUVECs and MSCs were seeded into 3 mg/ml 3-dimensional collagen hydrogels (TeloCol – 6, Advanced Biomatrix, Cat#. 5225-50ml) at a cell concentration of 250,000 cells/ml using the hanging drop technique [50]. In brief, HUVECs and MSCs were resuspended in EGM-2 and DMEM, respectively, and mixed 1:1 with a cold neutralized collagen type I solution. 10 µl drops of the mixture were placed onto the lid of a 90 mm Petri dish on day 0. The lid was then inverted carefully and placed in a humidified 37 °C incubator for 15 min for gel polymerization. Fully polymerized hydrogels were placed into cell culture wells containing control or MMC culture medium. After 2 days of culture, the collagen gels were fixed with 4% PFA. Collagen hydrogel and cells were imaged as described above in the *Microscopy* section. Gel size and average fluorescent intensity were quantified using ImageJ software (https://imagej.nih.gov/ij/).

### Rheology measurements

Cell-free collagen hydrogels with a final concentration of 3 mg/ml were polymerized as mentioned before, transferred to control medium or MMC containing medium for 2 days before undergoing rheology tests by using a rheometer (Malvern Kinexus Lab + Plate Package 20 N, KNX5, NETZSCH-Gerätebau GmbH). The viscoelasticity of the gel was measured at 37˚C at 1 Hz frequency and 0.1% shear strain.

### Statistical analysis

At least three independent biological runs with at least three replicates each were performed for each experiment. Statistical analysis of band intensity on Western blot, permeability coefficient, staining mean intensity, cellular contractility assay and MVNs quantification were calculated by Student’s t-test. Holm-Sidak test was performed for multiple comparisons, and p-values below 0.05 were considered statistically significant. The analysis was performed using GraphPad Prism v8.0 (GraphPad Software, San Diego, CA, USA, www.graphpad.com).

## Results

### MMC stabilized MVNs in microfluidic devices

Using a previously established design, consisting of three channels separated by permeable partitions (Fig. 1A), HUVECs and human bone marrow derived MSCs [51, 52], purposed as pericytes, were seeded in a fibrinogen solution into the center channel (Fig. 1B). The co-supplemented thrombin facilitated the formation of a stable fibrin hydrogel within minutes, which served as a 3D matrix for the embedded cells. No protease inhibitors were added to avoid interference with the biological processes within the devices. As reported previously [23], cells re-arranged into tubule-like structures within 2 days and formed interconnected MVNs (Fig. 1C). One day after cell seeding, MMC was optionally introduced as a supplemental treatment to the culture medium into the microfluidic devices. Cells not exposed to MMC were utilized as the control. We utilized an established MMC cocktail [39] based on a mixture of two Ficoll macromolecules (70 kDa and 400 kDa) dissolved in cell culture medium at a previously optimized concentration [39]. It has been shown previously that an entropy-based synergy is created by a mixture of two different size populations of artificial crowders, providing small crowders with extra volume occupancy when in the vicinity of larger ones [38]. The established crowder cocktail has been shown to drive ECM deposition for various cell types in 2D [37, 39] and recently also in 3D [41, 49].

**Fig. 2:**
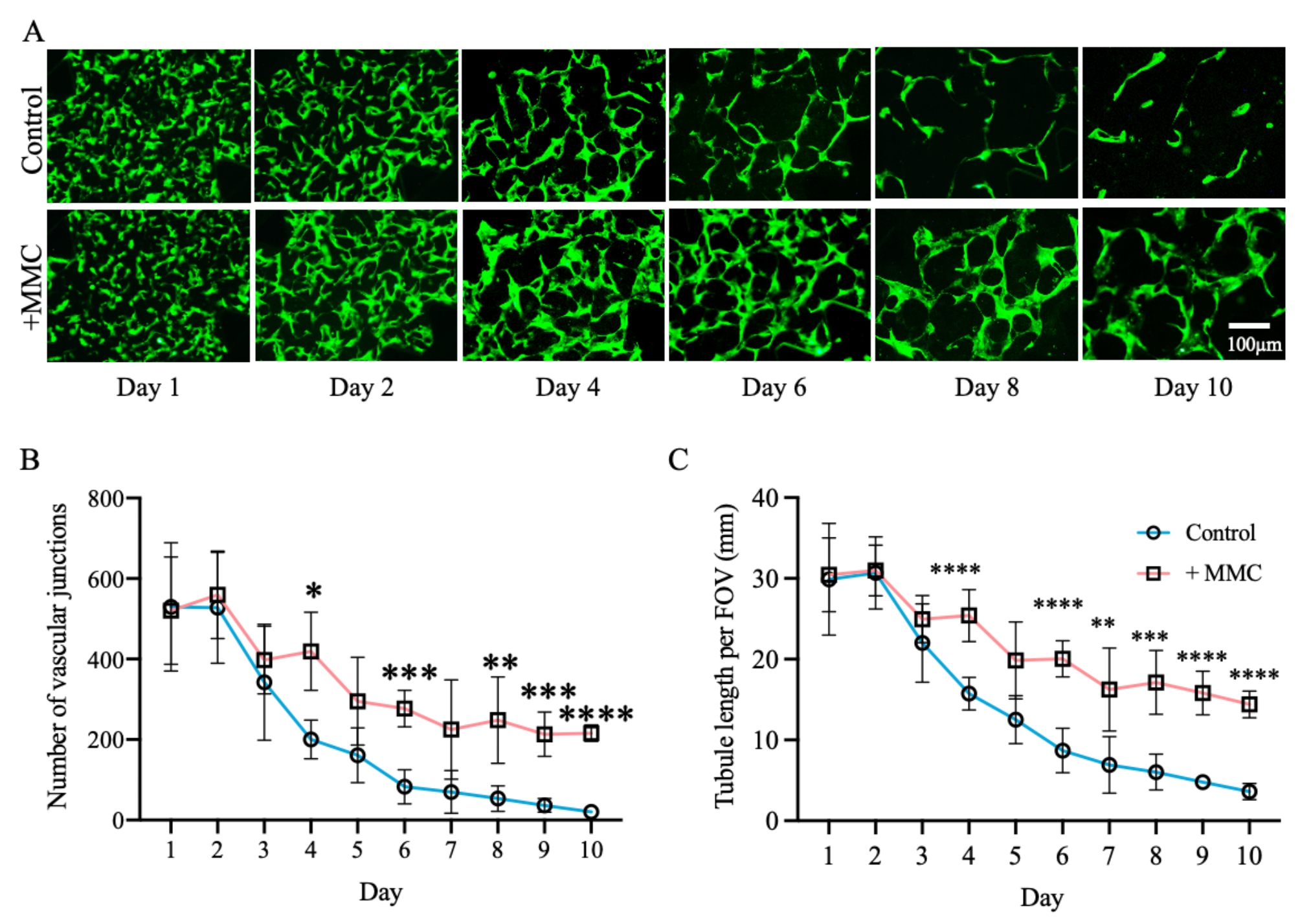
MMC stabilized MVNs in microfluidic devices. MVNs formed by GFP-expressing HUVECs cultured under control (no MMC) or crowded conditions (+ MMC) were continuously monitored for 10 days. (A) Representative pictures depicting fluorescent MVNs formed by GFP-expressing HUVECs at different time points. (B) Quantification of number of vascular junctions and (C) tubule length per field of view (FOV) using ImageJ angiogenesis analyzer plug-in. *, p < 0.05; **, p < 0.01; ***, p < 0.001; ****, p < 0.0001. n = 5 biological replicates. Scale bar = 100 μm

Utilization of green fluorescent protein (GFP)-expressing HUVECs allowed continuous tracking of the formation and regression of MVNs over time (Fig. 2). Early addition of the MMC cocktail to the microfluidic device did not affect the initial formation of an intricate MVN, as evident by the comparable morphology (Fig. 2A and S1), number of vascular junctions (Fig. 2B) and total tube length per field of view (FOV) (Fig. 2C) during the first 2–3 days of culture. Over time, MVNs under control (no MMC) and MMC-supplemented (crowded) conditions dynamically morphed and retracted into thicker tubular structures. Visually striking was the disintegration of MVNs into disjointed isolated structures from day 6 onwards under control conditions, whereas crowded MVNs remained continuous until the end of the study (day 10) (Fig. 2A). This was also reflected in the quantitatively assessed number of vascular junctions, as well as total length of tubular structures, which were significantly higher under supplementation of MMC from day 4 onwards (Fig. 2B, C).

### MMC promoted the formation and preservation of a vascular basement membrane

**Fig. 3:**
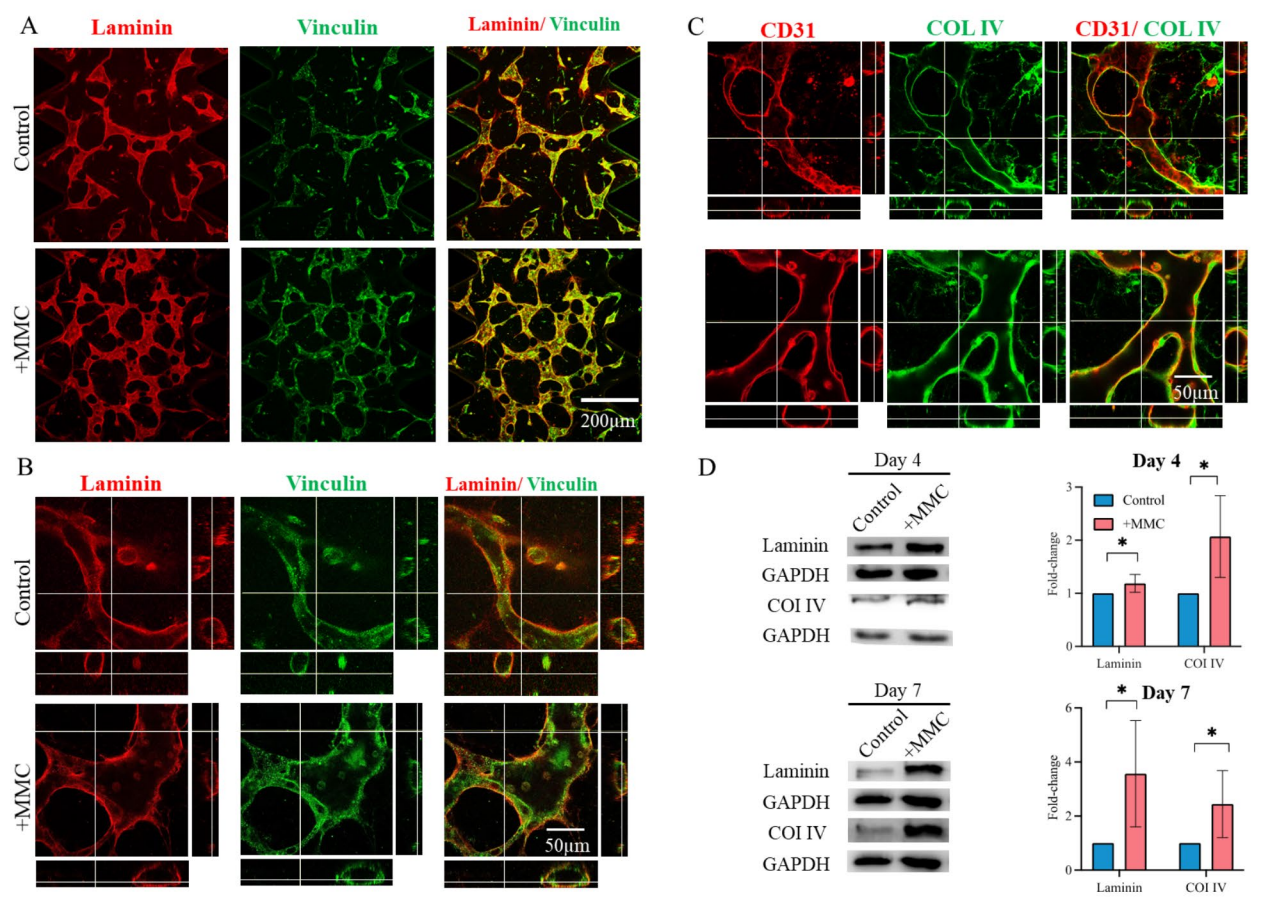
MMC promoted the formation of basement membrane enveloping microvascular hollow tubes. Day 4 MVNs were immunostained for basement membrane components, laminin and collagen IV, as well as the focal adhesion component, vinculin, and endothelial cell surface marker CD31. (A) Confocal microscopy images. Scale bar = 200 μm. (B,C) Confocal microscopy images including z-stack images displayed as orthogonal view. Locations of cross-sections are indicated by white lines and orthogonal cross-sections are displayed on the sides. Scale bar = 50 μm. (D) Western blot and densitometric band analysis of laminin and collagen IV normalized to their respective GAPDH levels of samples collected on days 4 and day 7. Protein levels are displayed as fold-change as compared to control. *, p < 0.05. n = 4 biological replicates

Since initial differences in MVN stability were noticeable on day 4 and became even more apparent by day 7, we investigated the localization of major basement membrane components by confocal microscopy on day 4 and quantified their abundance on days 4 and 7 by Western blot. As evident in the laminin staining (Fig. 3A), all microvascular structures were covered abundantly by basement membrane. Similarly, microvascular cells exhibited a robust expression of vinculin, suggesting that cells formed focal adhesions and interacted with their basement membrane. High magnification confocal z-stack analysis of formed microvasculature confirmed that the tubular structures were in the size range of capillaries (< 100 μm) [2] and exhibited hollow and circular lumina (Fig. 3B, C, S2). Furthermore, co-staining of focal adhesions (vinculin) and endothelial cell surface marker CD31 with basement membrane components laminin or collagen type IV, respectively, demonstrated that endothelial structures were enveloped in a tight sheath of basement membrane. These findings confirmed an apical-basal polarity of formed microvessels. Levels of basement membrane components were further evaluated by Western blot analysis of samples collected on days 4 and 7. For this, laminin and collagen IV levels were initially normalized to the respective levels of GAPDH (cell number, Fig. 3D) or CD31 (number of endothelial cells, Figure S3) of the same samples. Relative changes in basement membrane component levels were then displayed as fold-change to control culture (no MMC) conditions. As a result, a significant accumulation of collagen type IV and laminin in MMC-supplemented cultures was observed. This was irrespective of their levels being normalized to the number of total cells or endothelial cells (Fig. 3D, S3).

### MMC promoted the formation cell-cell junctions

**Fig. 4:**
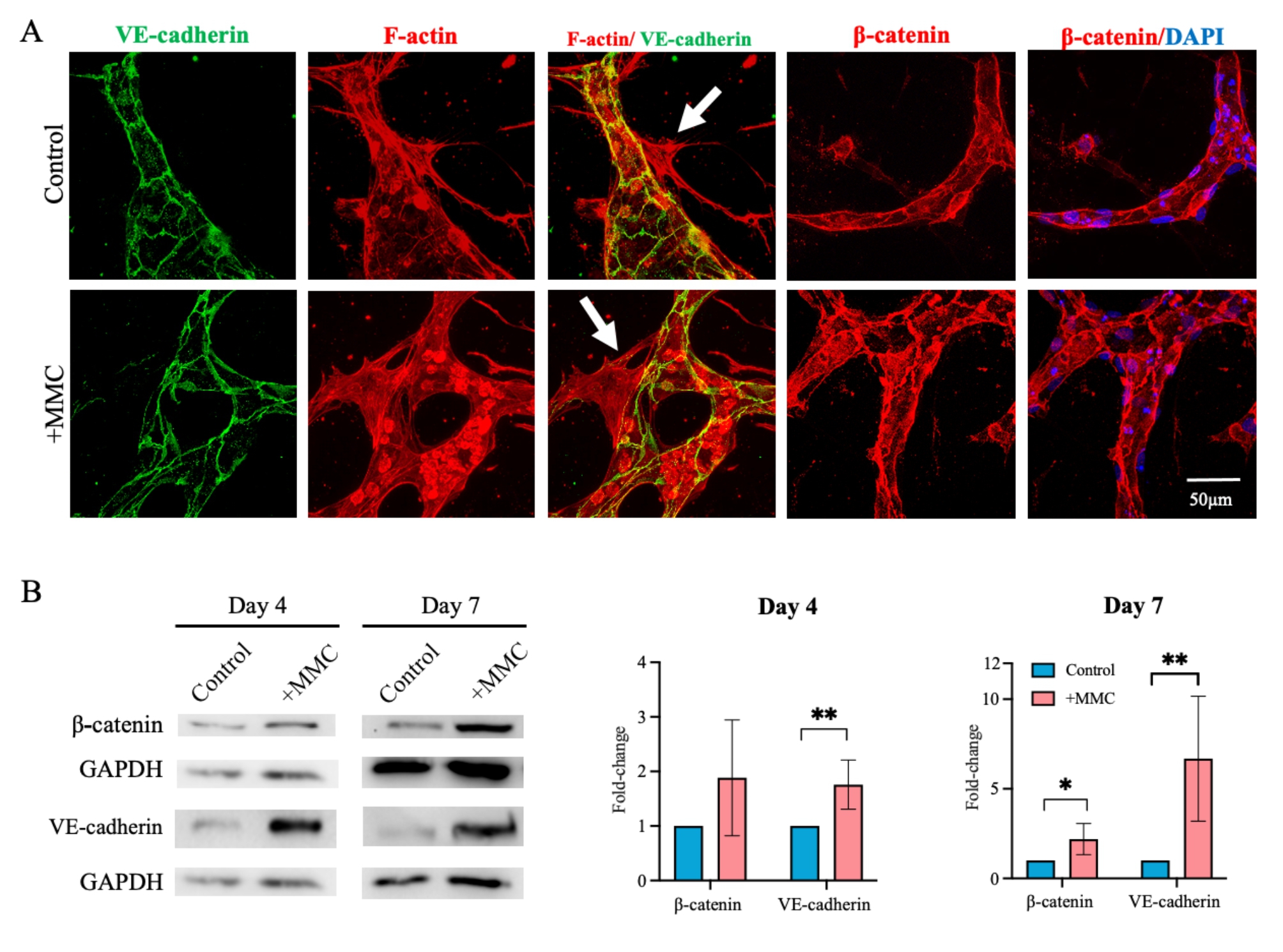
MMC promotes the formation of cell-cell junctions. (A) Representative maximum intensity projections of confocal z-stacks taken of MVNs grown under control or crowded (+ MMC) conditions and stained for DAPI and with phalloidin for F-Actin, as well as immunostained for adherens junction proteins, VE-Cadherin and β-catenin. Scale bar = 50 μm. (B) Western blot and densitometric band analysis of VE-cadherin and β-catenin normalized to the respective GAPDH levels of samples collected on days 4 and day 7. Protein levels are displayed as fold-changes as compared to control. *, p < 0.05; ***, p < 0.001. n = 4 biological replicates

Inter-endothelial cell-cell junctions are crucial for microvessel integrity and proper function. We thus investigated VE-cadherin and β-catenin localization and overall levels in MVNs cultured under crowded and control conditions (Fig. 4A). Maximum intensity projections of confocal slices taken from samples cultured for 4 days visualized junctional proteins sharply outlining endothelial cells in both conditions.

Furthermore, when endothelial cell-specific VE-cadherin was co-stained with F-actin, MSCs (negative for VE-cadherin) were identified taking up perivascular location (Fig. 4A, **white arrows**), as reported previously [23]. Western blot analysis of samples collected on days 4 and 7, revealed an abundance of VE-cadherin in MMC-supplemented cultures on both time points and a significant accumulation of β-catenin on day 7, irrespective of protein levels being normalized to the number of total cells (GAPDH levels, Fig. 4B) or endothelial cells (CD31 levels, Figure S3).

### MMC improved vascular barrier functions of MVNs

Vascular barrier function or low vascular permeability is dependent on tightly associated cell-cell junctions, as well as a properly assembled basement membrane and other factors [1]. As MMC resulted in the prominent accumulation of cellular junctional and basement membrane protein components in MVNs, we hypothesized that MVNs cultured under MMC might also exhibit a lower permeability and thus improved barrier functions.

**Fig. 5:**
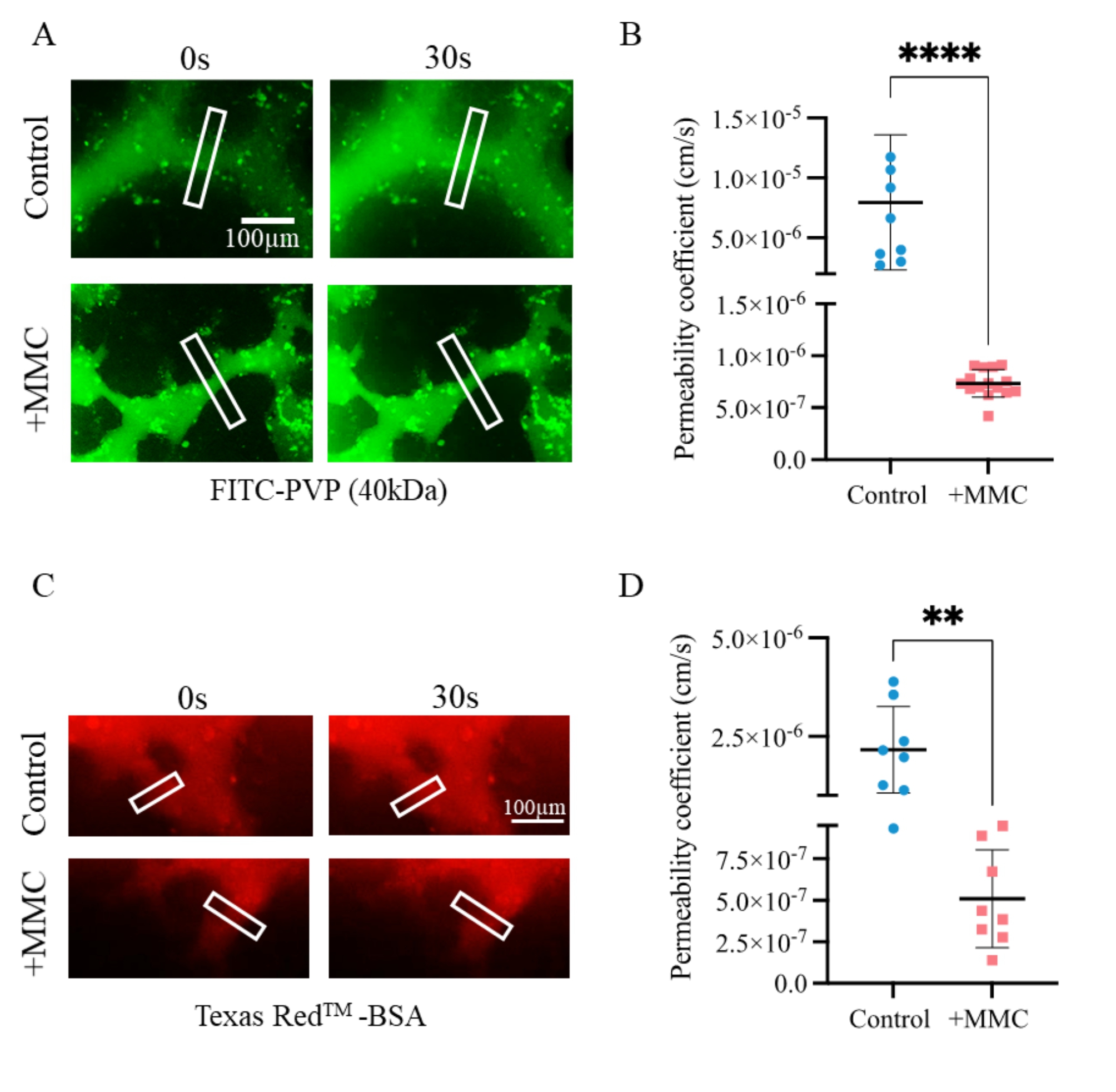
MMC decreased vascular permeability. MVNs cultured under crowded (+ MMC), and control conditions (no MMC) were perfused with FITC-PVP (40 kDa, A & B) or Texas Red^TM^-BSA (C & D), respectively, supplemented culture medium. (A) and (C) Representative frame from live cell imaging showing the perfusion of MVN from one medium channel to the other on day 4. Scale bar = 100 μm. (B ) and (D) Permeability coefficient of MVNs for FITC-PVP (40 kDa) and Texas Red^TM^-BSA, respectively, cultured under control and crowded conditions on day 4. **, p < 0.01; ****, p < 0.0001

In order to investigate this, HUVECs were additionally seeded into the medium channels on day 2 to form a continuous endothelial monolayer at the media-hydrogel interface. Seeded endothelial cells then anastomosed with the MVN of the center channel, thereby forming vascular openings to the medium channels by day 4. When culture medium was supplemented with fluorescein-5-isothiocyanate (FITC)-labelled polyvinylpyrrolidone (PVP, 40 kDa) or Texas Red^TM^-labelled bovine serum albumin (BSA) and added to one of the medium channels, live fluorescence microscopy clearly demonstrated that MVNs were perfusable from the medium channels under both conditions (Fig. 5A,C). However, vascular openings were clearly more abundant in MVNs cultured under crowded conditions (Figure S4), likely caused by the maintenance of well interconnected microvessels, resulting in more ready perfusability for MVNs grown under crowded conditions.

Vascular permeability can be measured through hindrance flux of solutes across the vessel wall [12]. Diffusive transport of FITC-PVP (40 kDa) and Texas Red^TM^-BSA across the microvascular wall was measured as a function of the change in fluorescence intensity in a defined volume in the perivascular space (hydrogel region) over time (imaging carried out every 15 s for 15 min). Assuming that microvessels exhibited circular tubular structures of less than 50 μm, the permeability coefficient was quantified, based on previously published methods [12]. Indeed, a permeability coefficient of 7.34 ± 1.31 × 10^− 7^ cm/s was obtained in 3D MVNs cultured under MMC, lower by one-order-of-magnitude as compared to 7.29 ± 5.71 × 10^− 6^ cm/s determined in control (no MMC) 3D MVNs for FITC-PVP (Fig. 5C). The current permeability coefficient determined for MVNs under crowded conditions is therefore in the same order of magnitude as for same size macromolecules in rat venular vessels *in vivo* (1.37 ± 0.26 × 10^− 7^ cm/s) [53], suggesting that crowded MVNs exhibited better vascular barrier function, comparable to physiological levels. Similarly, a permeability coefficient of 5.10 ± 2.95 × 10^− 7^ cm/s was obtained in 3D MVNs cultured under MMC, lower by one-order-of-magnitude as compared to 2.16 ± 1.10 × 10^− 6^ cm/s determined in control (no MMC) 3D MVNs for Texas Red^TM^-BSA (Fig. 5D). Hence, the current permeability coefficient determined for MVNs in the presence of MMC is in the same order of magnitude as that of post-capillary rat venules *in vivo* [54, 55].

### MMC reduced cellular contractility

One of the major mechanisms by which MVNs regress in microfluidic devices is via pruning and cellular retraction, both processes dependent on cellular contractility. Hence, we investigated whether MMC would also affect cytoskeleton assembly and cellular contractility in 3D.

**Fig. 6:**
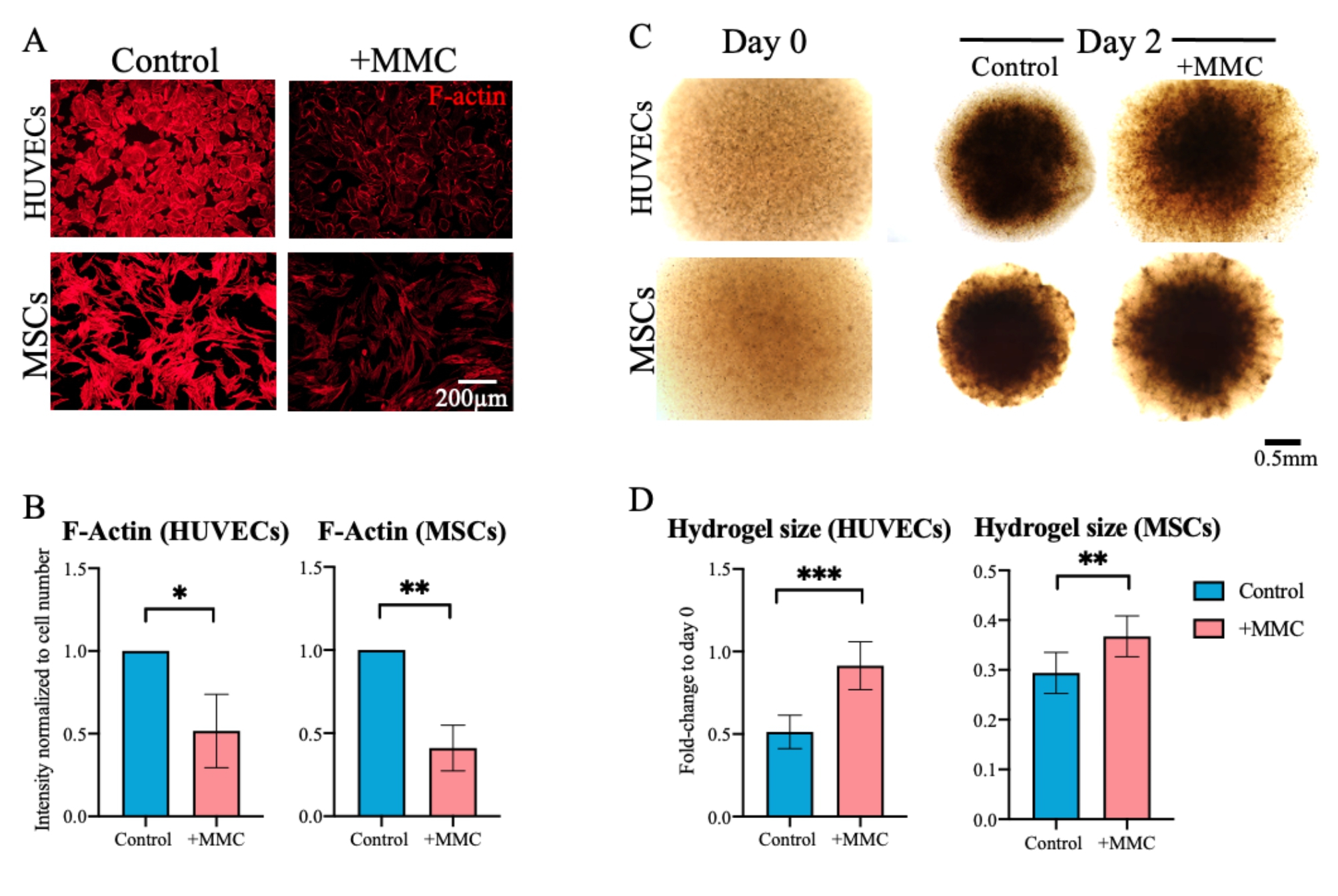
MMC reduced cellular contractility. (A) Fluorescence microscopy of phalloidin stained F-actin in 2D cultures of HUVECs and MSCs that were cultured in control (no MMC) or MMC medium for 2 days. Scale bar = 200 μm. (B) Quantification of fluorescence intensity of stained F-actin fibers normalized to cell number. (C) Functional cell contractility assay: HUVECs or MSCs were each seeded in spherical 3D collagen type I hydrogels on day 0 and cultured for 2 days under control or crowded (+ MMC) conditions. Phase contrast images were taken on day 0 and day 2. Scale bar = 500 μm (D) Diameter (size) measurements of cell-seeded hydrogels. Data are shown as relative fold-change of diameter as compared to day 0. *, p < 0.05; **, p < 0.01; ***, p < 0.001. n = 7 biological replicates

HUVECs and MSCs were allowed to attach overnight and then cultured for 2 days in 2D under crowded or control (no MMC) conditions, after which they were stained for filamentous actin (F-actin) by fluorescently-tagged phalloidin and imaged under identical conditions including laser intensity and exposure time. A striking reduction in F-actin staining intensity was observed under crowded conditions (Fig. 6A, B), suggesting a reduction in cytoskeletal force generation under MMC in both cell types. To test this hypothesis, each cell type was seeded into 3D spherical collagen type I hydrogels, which were allowed to fully polymerize to ensure comparable cross-linking density and thus mechanical properties, before being exposed to either culture medium for 2 days. Equilibrated initial hydrogel diameters (size) on day 0 and after 2 days of culture, were recorded and reduction in hydrogel size was calculated (Fig. 6C, D). To confirm that incubation of collagen hydrogels with or without MMC did not affect hydrogel mechanical properties, hydrogels free of cells underwent rheological analysis after 2 days of incubation in either culture medium. Indeed, a comparable storage modulus G’ of approximately 100 Pa for hydrogels incubated under control and MMC conditions was confirmed (Figure S5).

As reported previously [56, 57], cell-laden collagen hydrogels shrank in size, due to cellular contractility under both conditions. However, it is noteworthy that the size reduction was significantly more pronounced under control (no MMC) conditions, suggesting that both cell types were less contractile when cultured under MMC (Fig. 6C, D).

## Discussion

We report here the establishment of an easy and readily applicable and reproducible approach to stabilize and improve the functionality of 3D MVNs in microfluidic devices, which can be applied in a wide range of scenarios and laboratories. This protocol works without the previously necessary addition of auxiliary cells (fibroblasts) [20, 24], protease inhibitors [22, 27–29] or high doses of growth factors [23].

A major limitation of such *in vitro* engineered MVNs has been the limited stability and fast retraction of the formed microvessels under standard culture conditions [23]. Although there are a few studies that have reported longer lasting stabilities, some of us and many others have experienced that this is highly dependent on the lot of endothelial cells used, with the majority of MVNs retracting immediately upon formation. This characteristic largely limits the studies that are performed using MVNs in microfluidic devices. Strategies addressing this have included the introduction of large numbers of tertiary cells (fibroblasts) [20] and even immortalization of well performing cell types [30]. However, such approaches introduce another level of complexity, potentially changing the biological processes involved, and are not available in all research laboratories. Hence, there is a need for a more simple and straightforward approach to stabilize MVNs that is applicable to a broad variety of experimental set-ups.

The MMC cocktail utilized here can be directly dissolved in the culture medium and is well established, thus enabling a simplified approach to stabilize MVNs. The neutral, highly branched, hydrophilic Ficoll macromolecules used in the protocol are epichlorohydrin cross-linked poly-sucrose polymers and are commercially available [58]. They are widely used for gradient separation of hematopoietic stem cells and MSCs [58, 59], as well as freezing of *in vitro* fertilized eggs [60]. Ficoll is a clinically approved material in transfusion medicine and *in vitro* fertilization. Moreover, Ficoll macromolecules have been shown over the years to be compatible even with sensitive cell types and are thus not likely to interfere with cellular processes within the microfluidic devices. It is therefore not unexpected that the initial formation of MVNs was not affected. MVNs cultured under MMC exhibited hollow and circular lumen and small vessel diameter, apical-basal polarity with MSCs taking up perivascular locations and thus acting as pericytes and a vessel permeability comparable to *in vivo* observations, thus closely resembling a capillary bed.

The results presented here are also in line with those from a previous study investigating plasma expanders in a hydrogel-molded microvessel, which exhibited vessel stabilizing effects, improved VE-cadherin expression and reduced focal leaks [61]. Since the plasma expander was 3% dextran (70 kDa, a neutral carbohydrate macromolecule), it might have acted as macromolecular crowder.

The observed improved stability and functionality of MVNs under MMC likely results from a combination of factors, including better preserved basement membrane, cellular junctions, as well as reduced cell contractility, leading to reduced vascular retraction. Indeed, it has been postulated that the balance between cellular tension and adhesive forces regulates endothelial barrier function [62]. Laminin, an integrin ligand and major component of the vascular basement membrane, is highly enriched under MMC, while tightly sheathing endothelial tubular structures. A previous study suggested that laminin stabilizes vascular networks through inhibition of lumen expansion and tubular morphogenesis [63, 64]. Interaction of endothelial cells with basement membrane components such as laminin was also previously shown to regulate VE-cadherin localization and endothelial barrier function [65]. Interestingly, VE-cadherin was also highly enriched under MMC and previously reported to be crucial for vessel integrity [66, 67] by stabilizing endothelial junctions. Furthermore, VE-cadherin was demonstrated to bind to intracellular β-catenin and to interact with actin-binding proteins, such as vinculin, before anchoring this complex to the cytoskeleton, thereby controlling vessel permeability [68]. Hence, improved formation of cell-cell junctions under MMC is likely responsible for the improved vascular barrier functions.

It is noteworthy that changes in VE-cadherin-mediated cell-cell adhesion and integrin-mediated cell-basement membrane adhesion coordinately affect the physical and mechanical re-arrangement of the endothelial cells. Work by others demonstrated that VE-cadherin signaled through RhoA, thereby increasing cellular stress fiber formation and thereby resulting in cellular contractility and a denser focal adhesion formation [69]. This finding, however, could not be confirmed in our experimental set-up, as we observed a clear accumulation of VE-cadherin and focal adhesion ligands under MMC, while stress fiber formation and cellular contractility were decreased. Hence, MMC seems to be highly advantageous, as it promotes cell-matrix and cell-cell interactions while decreasing cellular contractility, thereby improving MVNs stability and functionality. This favored balance of adhesive forces to cellular tension is suggested to be the main driver of the improved endothelial barrier functions observed here. Other factors and conditions that have been reported to further improve endothelial barrier functions include culture under shear stress and/or hypoxia, as well as choosing the appropriate cell type, such as brain specific endothelial cells, pericytes and astrocytes to model the blood-brain-barrier [70]. We postulate that by introducing MMC into these cultures, MVN functionality and resemblance to physiological conditions can be further improved.

## Conclusion

In summary, by integrating two engineering core technologies, based on microfluidic MVNs and MMC, we have provided a reliable and flexible approach to stabilize engineered microvessels and improve their functionality, thereby enabling a closer resemblance to physiological conditions. Hence, utilization of MMC in MVN enables to engineer vascularized physiologically relevant microphysiological systems and micro-tissues on-a-chip, as well as potentially open avenues to engineer larger vascularized tissues for regenerative medicine.

## Electronic supplementary material

Below is the link to the electronic supplementary material.

Supplementary Material 1

## Data Availability

Data will be made available upon reasonable request to the corresponding author.

## Abbreviations

BCA: Bicinchoninic acid
BSA: bovine serum albumin
CAD: computer aided designs
CDI: carbonyldiimidazole
DMEM: Dulbecco’s Modified Eagle Medium
DMSO: Dimethyl sulfoxide
ECM: extracellular matrix
EGM-2: Endothelial Growth Medium-2
FBS: fetal bovine serum
FITC: fluorescein-5-isothiocyanate
FL: fluorescein
FOV: field of view
FVO: fractional volume occupancy
F-actin: filamentous actin
GFP: green fluorescent protein
HUVECs: human umbilical cord endothelial cells
MMC: macromolecular crowding
MSCs: mesenchymal stem cells
MVNs: microvascular networks
PBS: phosphate buffered saline
PDA: propylenediamine
PDMS: polydimethylsiloxane
PFA: paraformaldehyde
PVP: Polyvinylpyrrolidone
P/S: penicillin and streptomycin
