# Supplementary material for: Stabilization and improved functionality of three-dimensional perfusable microvascular networks in microfluidic devices under macromolecular crowding
Source: Biomater Res. 2023 Apr 19;27:32. doi: 10.1186/s40824-023-00375-w (PMC10116810; doi:10.1186/s40824-023-00375-w)
Supplement: Supplementary file 1 — Supplementary Material 1 [file 40824_2023_375_MOESM1_ESM.docx]

**Supplementary information**

**Stabilization and Improved Functionality of Three-dimensional Perfusable Microvascular Networks in Microfluidic Devices under Macromolecular Crowding**

*Ho-Ying WAN ^1,2^, Jack Chun Hin CHEN^3^, Qinru XIAO^3^, Christy Wingtung WONG^1,2^, Boguang YANG^4^, Benjamin Cao^5,6^, Rocky S. TUAN^1,2^, Susan K. NILSSON^5,6^, Yi-Ping HO^3^, Michael RAGHUNATH^7^, Roger D. KAMM^8^, Anna BLOCKI ^1,2,4,9,*^*

^1^ Institute for Tissue Engineering and Regenerative Medicine, The Chinese University of Hong Kong, Hong Kong SAR

^2^ School of Biomedical Sciences, Faculty of Medicine, The Chinese University of Hong Kong, Hong Kong SAR

^3^ Department of Biomedical Engineering, Faculty of Engineering, The Chinese University of Hong Kong, Hong Kong SAR

^4^ Department of Orthopaedics & Traumatology, Faculty of Medicine, The Chinese University of Hong Kong, Hong Kong SAR

^5^ Biomedical Manufacturing Commonwealth Scientific and Industrial Research Organisation (CSIRO), Melbourne, Australia

^6^ Australian Regenerative Medicine Institute, Monash University, Melbourne, Australia

^7^ Institute for Chemistry and Biotechnology, Zurich University of Applied Sciences, Wädenswil, Switzerland.

^8^ Department of Biology and Mechanical Engineering, Massachusetts Institute of Technology, Cambridge, MA, USA

^9^ Center for Neuromusculoskeletal Restorative Medicine (CNRM), Hong Kong SAR

* Corresponding author: Anna.Blocki@cuhk.edu.hk


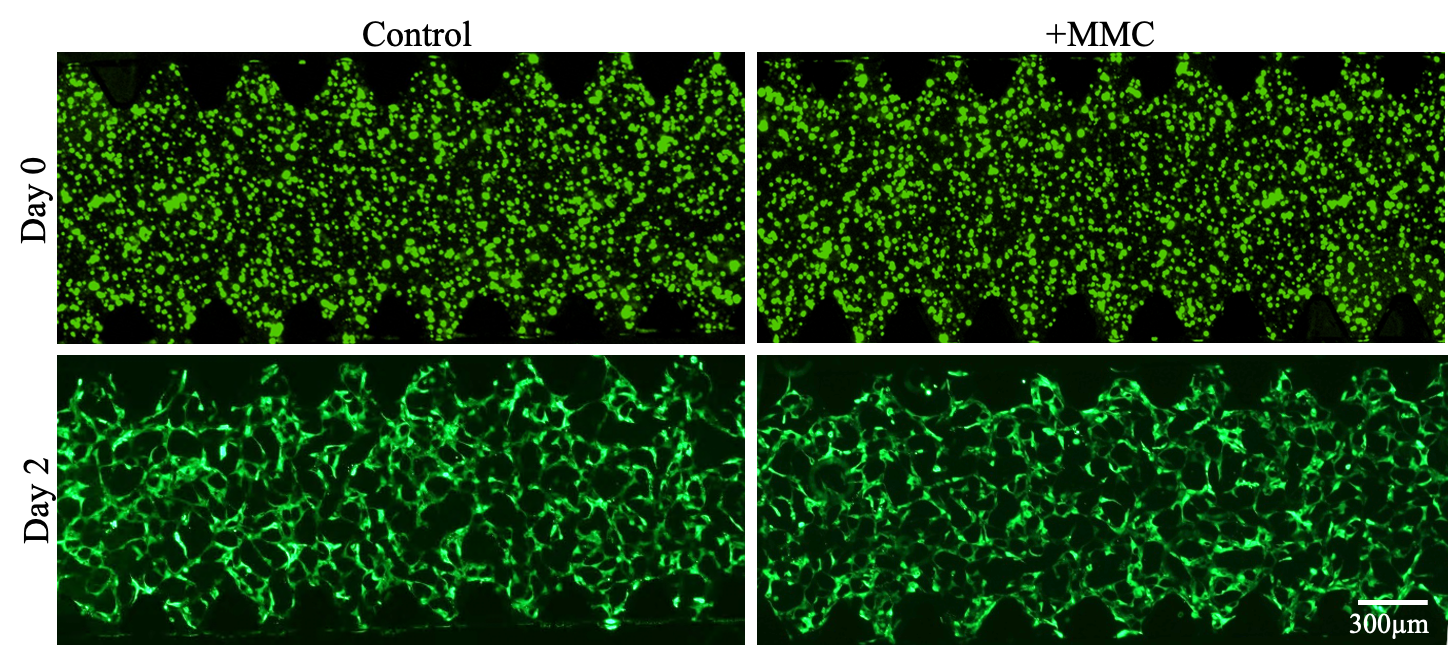


Supplementary figure S1. Representative pictures depicting fluorescent MVNs formed by GFP-expressing HUVECs on day 0 and day 2.


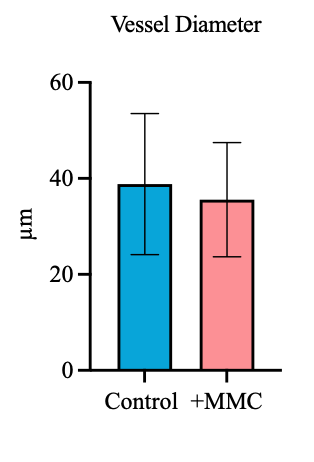


Supplementary figure 2. Quantification of vessel diameter in control and MMC treated MVNs on day 4.


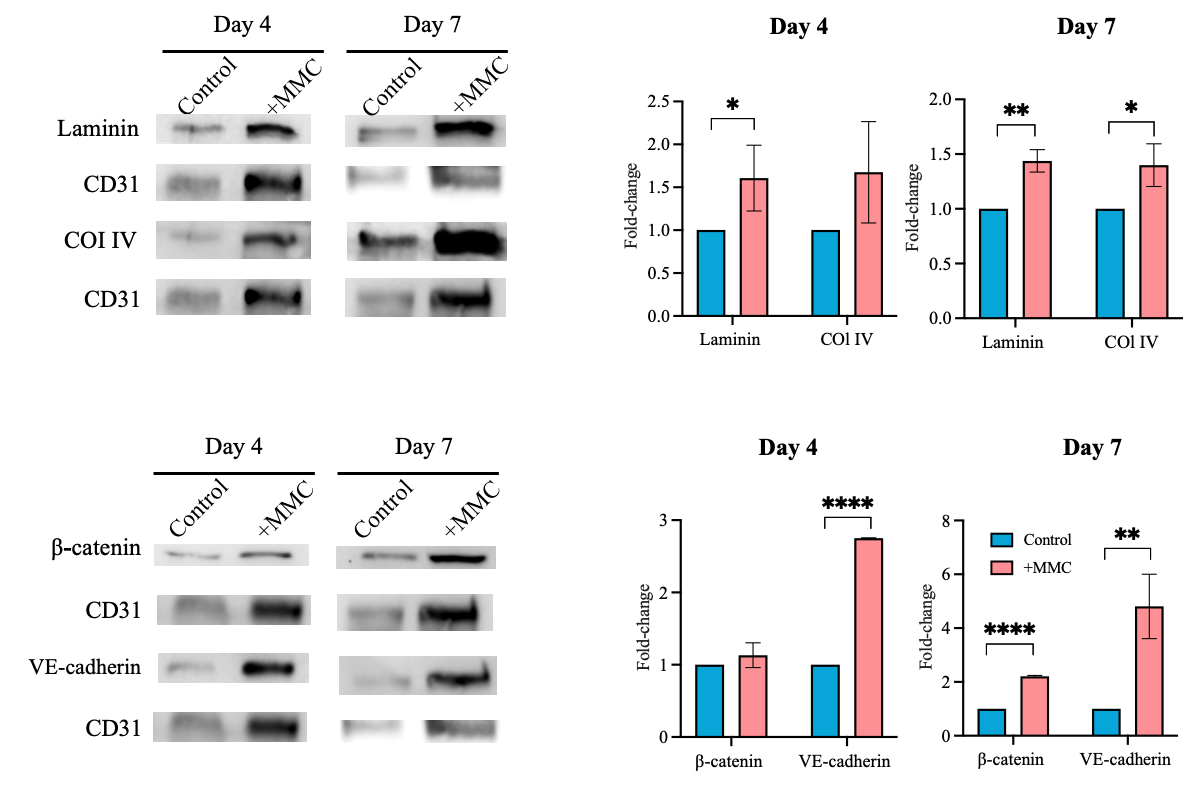


Supplementary figure S3. Western blot and densitometric band analysis of laminin, collagen IV, β-catenin and VE-cadherin normalized to their respective CD31 levels in samples collected on day 4 and 7. Protein levels are displayed as fold-change as compared to control culture conditions. **, p < 0.01, ****, p < 0.0005. n = 3 biological replicates.

Supplementary figure S4. Quantification of number of vessel openings to the media channels in control and MMC treated MVNs on day 4. ****, p < 0.0005

Supplementary figure S5. Storage modulus, G’ of collagen gels incubated in control or MMC containing medium for 2 days.
